# Fyn‐binding protein ADAP supports actin organization in podocytes

**DOI:** 10.14814/phy2.13483

**Published:** 2017-11-30

**Authors:** Zhenzhen Wu, Natalya A. Blessing, Jeffrey S. Simske, Leslie A. Bruggeman

**Affiliations:** ^1^ Department of Medicine and Rammelkamp Center for Education and Research MetroHealth Medical Center Case Western Reserve University School of Medicine Cleveland Ohio

**Keywords:** Cell junction, podocyte, proteinuria

## Abstract

The renal podocyte is central to the filtration function of the kidney that is dependent on maintaining both highly organized, branched cell structures forming foot processes, and a unique cell‐cell junction, the slit diaphragm. Our recent studies investigating the developmental formation of the slit diaphragm identified a novel claudin family tetraspannin, TM4SF10, which is a binding partner for ADAP (also known as Fyn binding protein Fyb). To investigate the role of ADAP in podocyte function in relation to Fyn and TM4SF10, we examined ADAP knockout (KO) mice and podocytes. ADAP KO mice developed glomerular pathology that began as hyalinosis and progressed to glomerulosclerosis, with aged male animals developing low levels of albuminuria. Podocyte cell lines established from the KO mice had slower attachment kinetics compared to wild‐type cells, although this did not affect the total number of attached cells nor the ability to form focal contacts. After attachment, the ADAP KO cells did not attain typical podocyte morphology, lacking the elaborate cell protrusions typical of wild‐type podocytes, with the actin cytoskeleton forming circumferential stress fibers. The absence of ADAP did not alter Fyn levels nor were there differences between KO and wild‐type podocytes in the reduction of Fyn activating phosphorylation events with puromycin aminonucleoside treatment. In the setting of endogenous TM4SF10 overexpression, the absence of ADAP altered the formation of cell‐cell contacts containing TM4SF10. These studies suggest ADAP does not alter Fyn activity in podocytes, but appears to mediate downstream effects of Fyn controlled by TM4SF10 involving actin cytoskeleton organization.

## Introduction

The slit diaphragm is a specialized cell junction that forms between foot processes of adjacent podocytes and is a major component of the filtration apparatus of the kidney. Its formation is a multi‐step process during development, beginning as a simple cadherin‐based junction that migrates to the basal cell surface and remodels into the mature structure containing the many prototypic proteins of the slit diaphragm including Nephrin, Podocin, CD2AP, and Neph‐1 (Reeves et al. 1978; Huber and Benzing 2005; Garg et al. 2007; Patrakka and Tryggvason 2010). Defects in or loss of these key slit diaphragm proteins are known to cause many genetic and acquired kidney diseases, and underscores the critical contribution of the podocyte‐produced components of the filtration barrier to kidney function (Grahammer et al. 2013).

Our recent studies investigating the early molecular events in the formation and assembly of the slit diaphragm identified a novel tetraspannin, TM4SF10 (gene name: *Tmem*47), as participating in the development of this unique cell junction (Bruggeman et al. 2007; Azhibekov et al. 2011; Dong and Simske 2016). TM4SF10 is a 20 kDa cell junction protein in the Claudin/PMP 22/EMP family (Bruggeman et al. 2007), and is one of the few claudin family proteins known to be expressed in podocytes (Yu 2015). TM4SF10 is highly expressed during kidney development, being transiently expressed at the basal‐most region of podocyte precursors in comma and s‐shaped bodies, but is absent by the capillary loop stage. Although its expression is absent in adult glomeruli, it can be re‐expressed in mature podocytes during glomerular injury repair processes (Bruggeman et al. 2007; Azhibekov et al. 2011). In *Caenorhabditis elegans*, the TM4SF10 ortholog VAB‐9 has been well characterized. VAB‐9 has a transient but essential role organizing contractile F‐actin filaments to insert into the developing adherens junction complex of the worm epidermis, and has a redundant role in cell adhesion (Simske et al. 2003). In cultured MDCK cells, TM4SF10 also has a role in regulating cell junction dynamics (Dong and Simske 2016).

To determine the functional role of TM4SF10 in the mammalian kidney, we previously screened a murine fetal kidney library to identify TM4SF10 interacting partners and identified the large cytoplasmic adapter protein ADAP, also known as the Fyn binding partner Fyb (gene name: *Fyb*) or Slap‐130 (Azhibekov et al. 2011). ADAP, like the slit diaphragm protein CD2AP (Shih et al. 1999), was originally described in T cells. ADAP is part of the T‐cell receptor signaling complex where it associates with numerous effector proteins involved in actin dynamics, integrin binding, and NF‐*κ*B signaling (Griffiths and Penninger 2002; Medeiros et al. 2007; Menasche et al. 2007; Burbach et al. 2008, 2011; Wang and Rudd 2008; Srivastava et al. 2010; Pauker et al. 2011). Upon recruitment to the T‐cell receptor, ADAP becomes tyrosine phosphorylated, creating binding sites for the SH2 domains of Fyn and SLP‐76, another adapter protein which binds Nck (although there is evidence Nck binds ADAP directly (Sylvester et al. 2010)). ADAP is an intriguing TM4SF10 interacting protein since it consolidates multiple processes in actin cytoskeleton remodeling during integrin binding at the immunologic synapse. Evaluating similar roles for ADAP in podocytes may shed light on the unique functions of podocyte cell‐cell and cell‐matrix interactions that govern slit diaphragm and podocyte foot process formation during development and foot process loss during disease pathogenesis.

Although our prior screening studies identified ADAP as a binding partner of TM4SF10 in the renal cells, the function of kidney‐expressed ADAP has not been previously examined. The goal of this study is to begin to investigate the role of ADAP in podocytes, and its function in kidney physiology by examining an ADAP knockout mouse model and ADAP knockout podocyte cell lines.

## Materials and Methods

### ADAP KO mouse model and KO podocyte cell lines

All animal studies were approved and conducted under the oversight of the Animal Care and Use Committee of Case Western Reserve University. The *Fyb* ^−/−^ global null mouse has been previously described and is on the C57Bl/6 background (Peterson et al. 2001). Standard breeding practices were used to maintain this line, and for all experiments including the aging study, mice were housed in a specific pathogen free (modified barrier) facility with microisolator caging in ventilated racks, handled with aseptic technique in laminar flow hoods, and given sterile ad libitum food and water.

To create ADAP knockout (KO) podocyte cell lines, *Fyb* ^−/−^ mice were intercrossed with the Immortomouse (Charles River Laboratories) which ubiquitously expresses a temperature sensitive SV40 large T antigen (tsA58). This temperature sensitive T antigen permits cell proliferation at reduced temperature (33°C, permissive conditions), but at physiologic temperature (37°C, nonpermissive conditions) the proliferative effect is eliminated and the cells resume a state of terminal differentiation. A homozygous male for the tsA58 transgene was intercrossed with a female *Fyb* ^−/−^ mouse, and the heterozygous F1 offspring were crossed to create F2 offspring, regenerating some homozygotes for the knockout allele. The F2 offspring were genotyped for presence of the tsA58 transgene using a PCR method previously described (Kern and Flucher 2005). Homozygosity for the *Fyb* ^−/−^ allele was verified using PCR primers as previously described (Peterson et al. 2001). Offspring that were positive for the tsA58 transgene that were *Fyb* ^+/+^ were also used for cell line development to create wild‐type (WT) cells for comparison. Glomeruli were isolated using the standard sieving method and primary podocyte outgrowths were cultured as previously described (Mundel et al. 1997). Cells were cloned by limiting dilution and podocyte phenotype was confirmed by expression of typical podocyte markers including Nephrin (*Nphs1*), Synaptopodin (*Synpo*), WT‐1 (*Wt1*), and Collagen IV*α*3 (*Col4a3*) by rt/PCR. Three clones each for both WT and KO podocytes were analyzed for experiments. For experiments, cells were used after culture for 7–10 at 37°C as previously described (Shankland et al. 2007).

Podocyte cultures were treated with puromycin aminonucleoside (“PAN”, Sigma) at doses as noted on figures as previously described (Rico et al. 2005). Cells were lysed for Western blotting or fixed with neutral formalin for immunostaining as previously described (Azhibekov et al. 2011). For cell attachment kinetics, podocytes were cultured on type I collagen‐coated plates for specified times, fixed in neutral formalin, and stained with 0.1% crystal violet. Final graphical data are a composite of all clones tested (performed three times) and data are reported as mean ± SD with statistical significance determined by unpaired *t* test; *P* > 0.05 were considered significant.

### Renal pathology and immunohistochemistry

**Figure 1:**
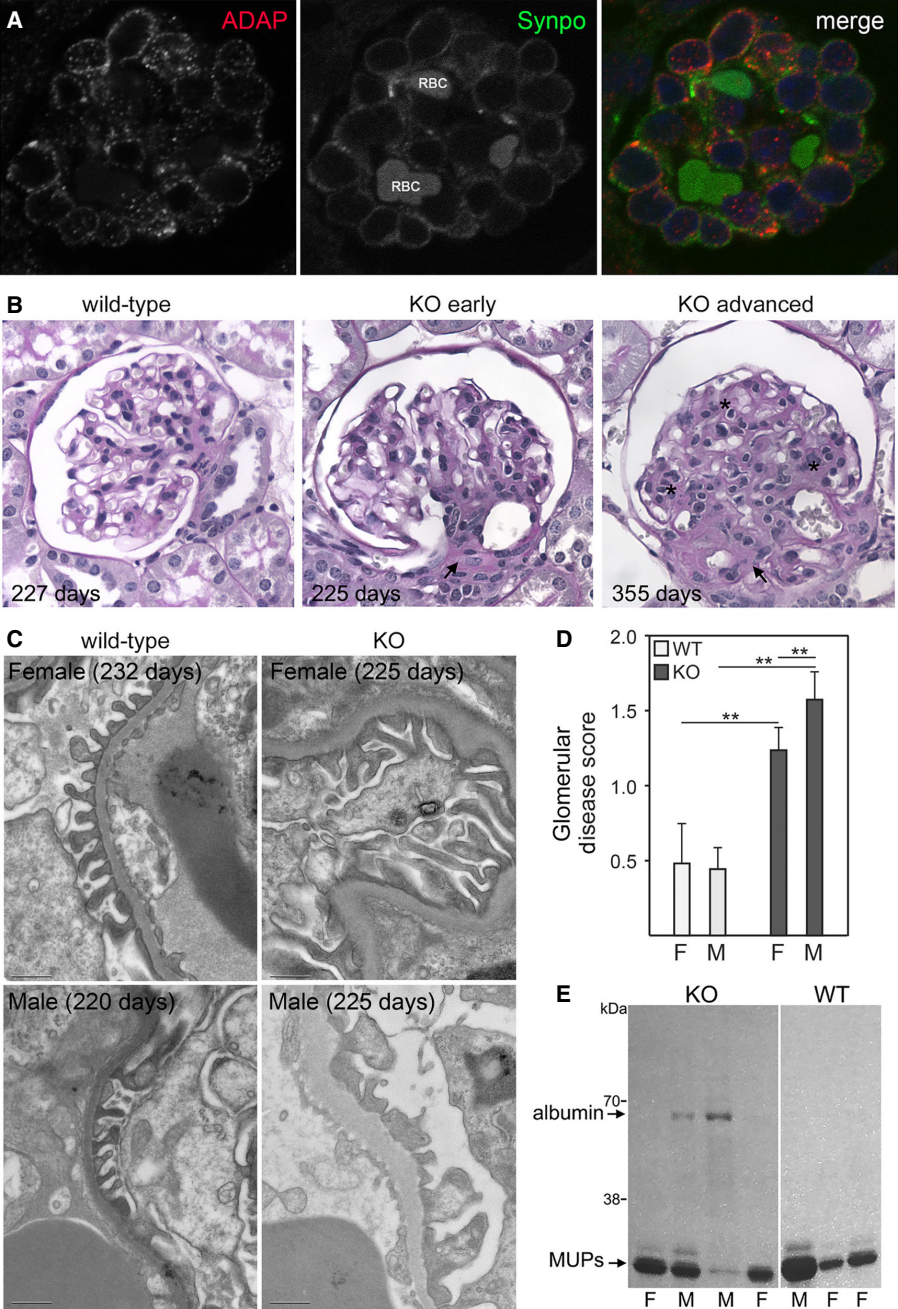
ADAP is expressed in podocytes and ADAP KO mice spontaneously developed a glomerular phenotype. (A) Immunofluorescence microscopy for ADAP expression in a newborn, wild‐type mouse kidney. ADAP expression was most abundant in developing podocytes at the capillary loop stage. (B) Histopathology (PAS stain) of age‐matched WT littermates and ADAP KO glomeruli showing initial lesions presenting as hyalinosis and progressing to advanced lesions exhibiting sclerosis and mesangial matrix expansion in the tuft. In our scoring system (0–4, see Methods), the WT panel would score = 0, the KO early panel would score = 2; and the KO advanced panel would score = 4. (C) Ultrastructural studies by transmission electron microscopy revealed glomerular basement membrane thickening and foot process widening and effacement. Age of mice is shown on image, scale bar = 1 *μ*m. (D) Quantification of histopathological changes. Glomerular lesions were significantly greater in KO mice at 1 year of age and more severe in male mice (**P <* *0.01). (E) Example of the proteinuria in 1 year‐old mice by Coomassie stained polyacrylamide gel. Proteinuria was more evident in male KO mice. Low molecular weight proteins are common and normal in the urine of mice (MUPS, major urinary proteins). Mean ages of mice in 200 day group: females 228 days and males 220 days. Numbers examined in the 200 day group were: KO female *n* = 9, KO male *n* = 3, WT female *n* = 6, WT male *n* = 5. Mean ages of mice in 1 year group: females 342 days and males 354 days. Numbers examined in the 1 year group were: KO female *n* = 8, KO male *n* = 6, WT female *n* = 7, WT male *n* = 3.

Kidney pathology was assessed using standard periodic‐acid Schiff (PAS) staining on formalin‐fixed, paraffin‐embedded 4 *μ*m sections. Transmission electron micrographs were obtained using standard methods on Karnovsky‐fixed, epon‐embedded sections. On light microscopy, glomeruli were scored for pathological changes using a scale of 0‐4 where 0 = normal, and abnormalities were scored as: 1 = hyalinosis only, changes restricted to glomerular hilum, 2 ≤ 50% segmental progression of sclerosis/mesangial matrix expansion, 3 ≥ 50% segmental changes, 4 = obsolete glomeruli. Examples of glomeruli with these scoring differences are shown in Figure 1. A minimum of 100 glomeruli in full sagittal sections were counted per animal. Percentage of glomeruli scoring abnormal (categories 1–4 above) were multiplied by their disease score and summed to generate a composite score, such that mice with 100% normal glomeruli would score zero and mice with 100% obsolete glomeruli would score four. Group means ± SD are reported and statistical differences were determined by unpaired *t* test.

### Immunostaining and Western blotting

Cells were cultured on type I collagen‐coated German 12 mm round glass coverslips at 10^4^ cells/well in 24‐well cluster plates. Mouse kidney tissue was formalin fixed, paraffin‐embedded, and 4 *μ*m kidney sections were processed using an antigen retrieval process as previously described (Madhavan et al. 2011). Immunostaining for tissue and cells used ADAP (Epitomics 2360‐1, 1:200), Synaptopodin (Biodesign, 1:10), Vinculin (Sigma, 1:100). For cultured cells, rhodamine‐conjugated phalloidin (1:1000 dilution, Molecular Probes) was used to detect actin structures. Western blotting was performed as previously described (Azhibekov et al. 2011). Antibodies and dilutions used included: focal adhesion kinase (“FAK”, Cell Signaling 1:1000); FAK‐pY^397^ (Cell Signaling, 1:1000); FAK‐pY^575/577^ (Cell Signaling, 1:1000); FAK‐pY^925^ (Cell Signaling, 1:1000); ADAP (Epitomics 2360‐1, 1:500); Fyn (Upstate, clone S1, 1:400); Src‐pY^418^ (Invitrogen, Carlsbad, CA, 1:1000, corresponding murine tyrosine is 421); Src‐pY^527^ (Cell Signaling, 1:1000, corresponding murine tyrosine is 532); TM4SF10 ((Dong and Simske 2016), 1:400) and Tubulin (Sigma, 1:8000). The specificity of the ADAP antibody was confirmed in immunohistochemistry by comparing immunostaining in the organs with highest ADAP expression (spleen, thymus, and lymph nodes) of WT and KO tissues and observed no staining in the KO tissues above the no primary antibody control. Western blots were repeated three times and band intensity was quantified with ImageJ.

## Results

### ADAP expression in normal mouse kidney

ADAP expression was evident in developing mouse kidney glomeruli at the capillary loop stage and co‐labeled cells that were positive for the podocyte marker Synaptopodin (Fig. 1A). Glomerular ADAP expression levels detected in adult normal mouse kidney were considerably lower in glomeruli, except for immune cells in vascular spaces (data not shown). This expression pattern of higher levels during glomerular development that wanes in the adult kidney was similar to the expression pattern of TM4SF10, which has highest during glomerular development, but absent in normal, adult glomeruli (Bruggeman et al. 2007).

### ADAP KO kidney phenotype

A mouse model of a global knockout of ADAP (ADAP KO or *Fyb* ^*−/−*^) has been previously described. These mice are viable with normal fecundity and have minor abnormalities in immune responses due to a partial disruption of integrin interactions between T cells and antigen presenting cells resulting in reduced signaling through the immunologic synapse (Peterson et al. 2001). A renal phenotype, however, was not been previously reported. Weaning age animals had no obvious histopathological changes, but a progressive glomerular pathology developed with age. These changes began as hyalinosis and progressed to focal and segmental sclerosis (Fig. 1B). On electron microscopy, there was evidence of foot process widening or effacement and basement membrane thickness irregularities in ADAP KO mice (Fig. 1C). Although it has been reported C57Bl/6 mice from some commercial suppliers can develop an age‐related kidney phenotype (Yabuki et al. 2006; Schmitt et al. 2009; Yang et al. 2010), the observed glomerular changes in the ADAP KO mice were histopathologically different, occurred in younger animals, and were significantly greater compared to age‐matched normal mice (Fig. 1D). Quantitative scoring of glomerular lesions indicated the pattern of pathology was clearly both focal (~50% of glomeruli were affected), and segmental (few fully obsolete glomeruli, see methods). Some mice >300 days of age also developed proteinuria, which was more common in male mice, but was not observed in age‐matched normal mice (Fig. 1E). This sex difference was evident in both the degree of histopathological changes and in proteinuria. Although male mice consistently exhibited low levels of albuminuria, female mice rarely developed albuminuria at 1 year of age.

### ADAP KO podocyte phenotype and effects on cell adhesion

**Figure 2:**
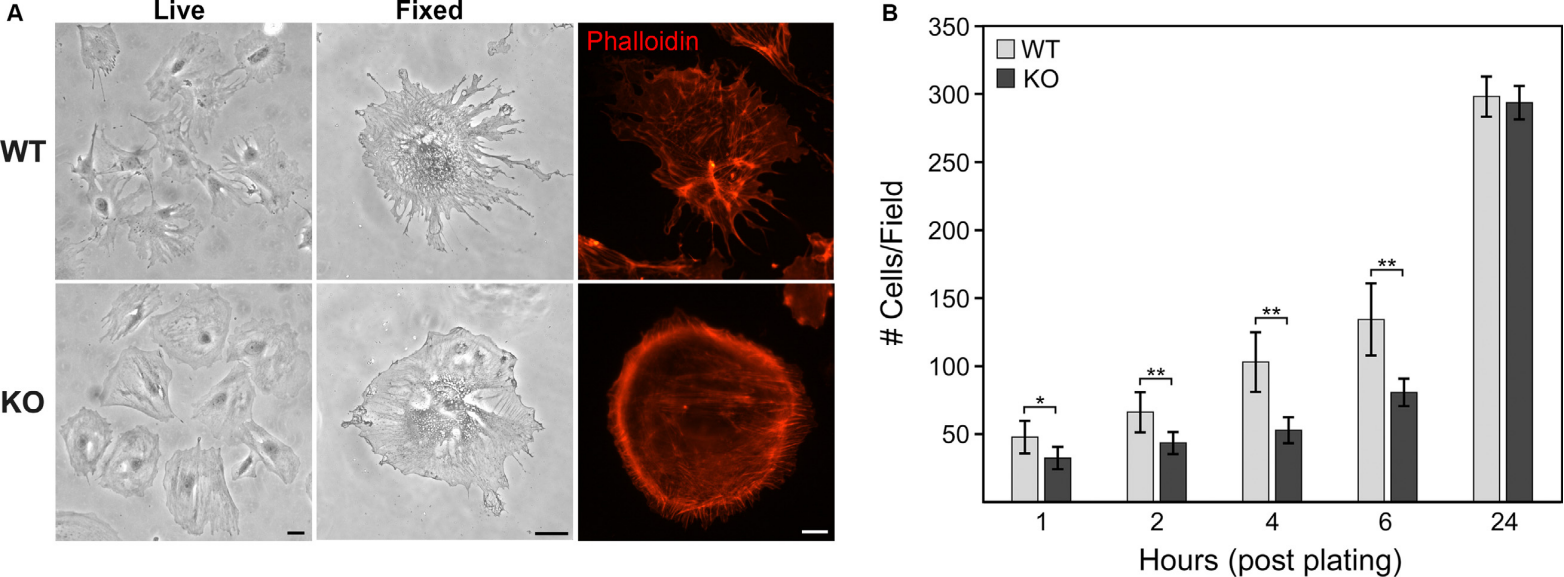
Cultured ADAP KO podocytes have altered morphology. (A) White light micrographs of live and fixed cultured podocytes and immunohistochemistry of actin cytoskeleton (Phalloidin) comparing WT and ADAP KO mice. KO had few long cellular protrusions typical of podocytes, with circumferential distribution of actin stress fibers. Scale bar = 25 *μ*m. (B) Attachment kinetics of WT and KO podocytes on collagen‐coated substrate. Although there was an initial delay in attachment of KO podocytes, by 24 h a similar number of cells were attached (*P <* *0.05, **P <* *0.01). [Color figure can be viewed at wileyonlinelibrary.com]

Multiple podocyte cell lines were established from ADAP KO and C57Bl/6 wild‐type (WT) mice by intercrossing with the Immortomouse and backcrossing F1s to recreate the *Fyb* ^*−/−*^ and *Fyb* ^*+/+*^ genotypes. Primary podocyte outgrowths from isolated glomeruli were cloned by limiting dilution and characterized for typical podocyte differentiation markers (Nephrin, WT‐1, Synaptopodin) by RT/PCR. Morphologically, ADAP KO podocyte had fewer of the long, branched extensions typical of normal podocytes (Fig. 2A). These protrusions contained actin, and F‐actin structures detected with phalloidin staining showed ADAP KO podocytes had a marked concentration of circumferential stress fibers compared to WT (Fig. 2A).

**Figure 3:**
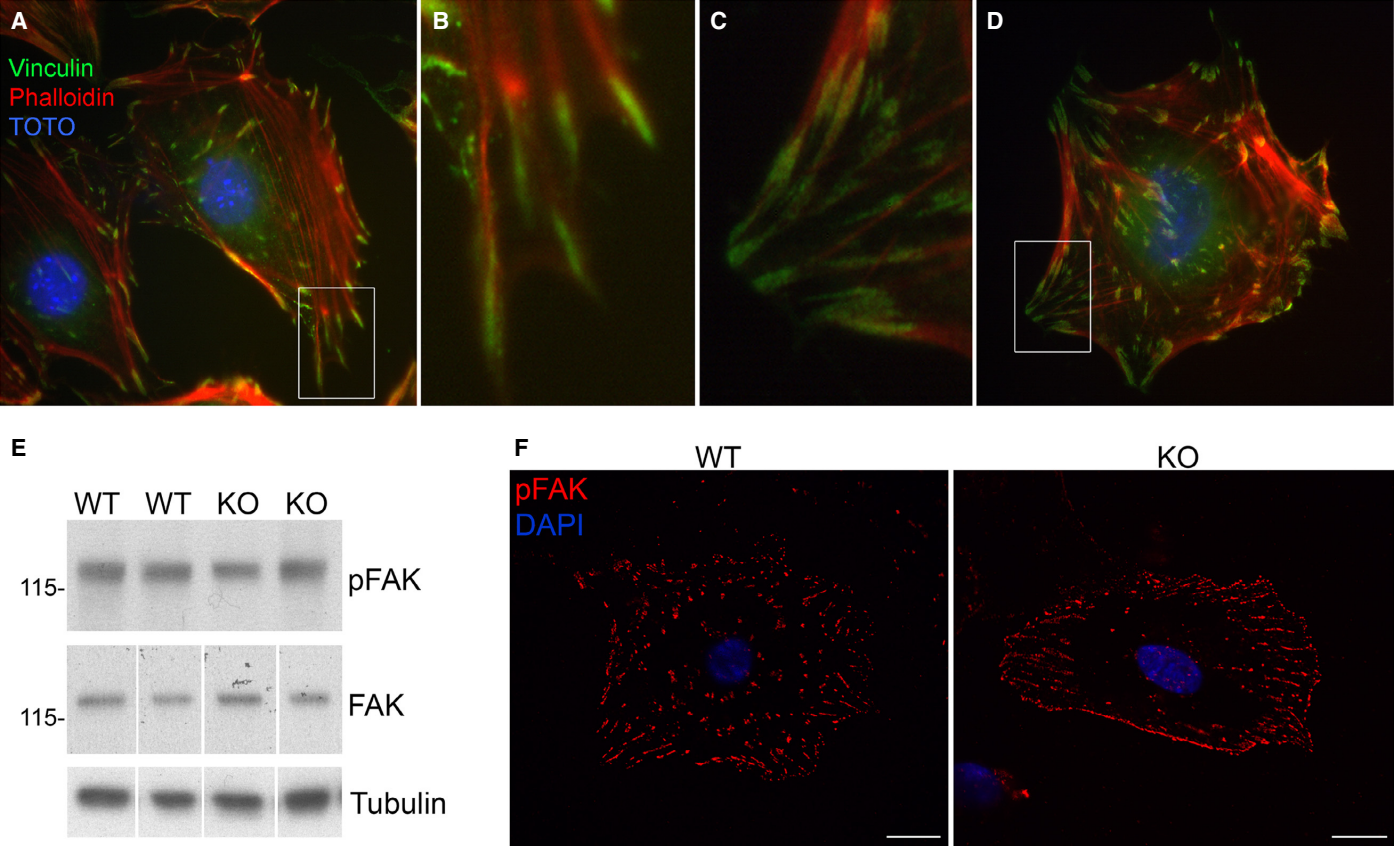
Formation of focal contacts are not different in KO cells. (A–D) Immunolocalization of focal contacts (vinculin) and actin cytoskeleton (phalloidin) in WT and KO podocytes. Insets (B, C) show details of distal actin filaments terminating in focal contacts at the cell perimeter. (E) Western blot of total FAK and FAK‐pY ^397^ in two WT and two KO clones, tubulin used as loading control. Quantification of blots is reported in text. (F) Immunofluorescence staining for FAK‐pY ^925^ in WT and KO podocytes. Scale bar=20 *μ*m.

The kinetics of cell adhesion of KO and WT podocyte lines were examined with an attachment time course. There was an initial delay in integrin‐dependent attachment (type I collagen coated surfaces) of KO podocytes compared to WT, however, by 24 h there was no significant difference in the final number of adherent cells (Fig. 2B). In attached cells, vinculin‐positive focal contacts similarly aligned at the tips of actin fibers in both KO and WT podocytes (Fig. 3A–D). However, since the KO cells lack the cellular protrusions typical of cultured podocytes, these focal contacts appeared blunted and aggregated at cell margins (Fig. 3C and D). Although focal contacts were distributed differently in the cells, there was no difference in focal adhesion kinase (FAK) levels between WT and KO podocytes by Western blotting (Fig. 3E). Phosphorylation of FAK at residue Y^329^ was similar between WT and KO cells and paralleled total FAK levels (quantification WT 0.71 ± 0.11 vs. KO 0.62 ± 0.08) as determined by Western blotting (Fig. 3E) and with a similar difference in distribution reflecting cell morphology differences (Fig. 3F). No differences were detected by both methods for downstream FAK phosphorylation events on Y^575/577^ or Y^925^ (data not shown). This suggested a similar ability to form focal adhesions although with a different distribution reflective of the cell morphology.

**Figure 4:**
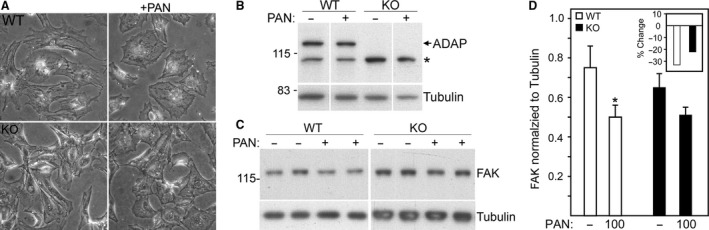
PAN treatment alters cell protrusions and focal contacts independent of ADAP expression. (A) Light micrographs of fixed cells showing typical loss of cellular protrusions in WT podocytes with PAN treatment, which is less evident in KO podocytes. (B) ADAP Western blot of WT and KO podocytes with and without PAN treatment showing ADAP levels are not altered by PAN treatment (* indicates nonspecific band). (C–D) FAK Western blot of WT and KO podocytes with and without PAN treatment (duplicates are shown). Untreated WT and KO podocytes had similar levels of FAK. PAN treatment of WT podocytes results in a significant reduction in FAK (*P <* *0.05), and PAN treatment of KO podocytes similarly trended to reduced FAK levels, but this was not significant. (D) Quantification of FAK bands in panel (C) inset shows percent change compared to untreated. Tubulin used as a loading/normalization control.

Puromycin aminonucleoside (PAN) is an accepted in vivo and in vitro method to study podocyte injury/repair events associated with proteinuria (D'Agati 2008). Short term treatment of WT and KO podocytes with PAN resulted in the typical retraction of cellular protrusions in WT cells, but this morphological change was less evident in KO cells since they had fewer cell protrusions initially (Fig. 4A). PAN treatment did not alter ADAP protein levels in either WT or KO cells (Fig. 4B). FAK protein levels were not different between untreated WT and KO cells, but with PAN treatment, FAK was reduced (33% reduction) in WT cells (Fig. 4C and D). The KO cells similarly responded to PAN treatment with reduced FAK levels (22% reduction), but this was not statistically significant (Fig. 4C and D), probably reflecting the fewer initial cell protrusions in the baseline KO cell morphology.

### ADAP effects on Fyn

**Figure 5:**
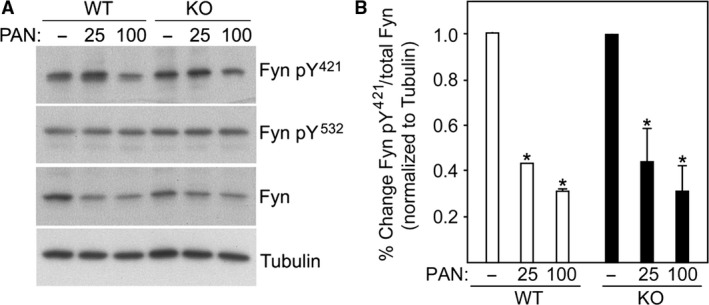
ADAP does not affect Fyn activation. (A) Western blot of untreated and PAN treated WT and ADAP KO podocytes for Fyn expression and levels of Fyn activating (pY ^421^) and inactivating (pY ^532^) phosphorylation events. (B) Quantification of band intensities of replicas indicated a similar PAN response in the Fyn activating pY ^421^ phosphorylation for both WT and KO podocytes. Band intensities normalized to tubulin with the untreated sampled set at 100% for each replica; *P <* *0.05 compared to untreated.

**Figure 6:**
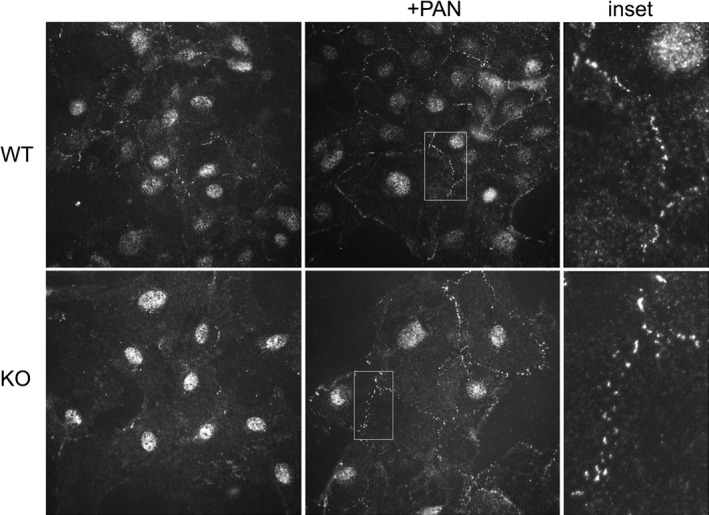
ADAP loss alters TM4SF10 localization at cell‐cell junctions. WT and KO podocytes were treated with PAN and immunostained for TM4SF10 expression. The nuclear staining of the TM4SF10 antibody is nonspecific (secondary antibody‐dependent). TM4SF10 expression in untreated cells was weak, with PAN treatment TM4SF10 expression was induced and concentrated at cell‐cell contacts. Inset: The distribution of TM4SF10 in cell‐cell contacts in the ADAP KO cells was discontinuous compared to WT cells.

The Src family kinase Fyn has been shown to be critical for podocyte slit diaphragm and foot process formation and maintenance (George and Holzman 2012). Since ADAP is a known Fyn binding protein, the role of ADAP in altering Fyn activity was examined in WT and KO podocytes. For these studies, Fyn phosphorylation of at tyrosine (Y)^421^, associated with kinase activation, and Y^532^ associated with kinase inhibition were examined by Western blotting (Fig. 5A and B). We have previously shown in MDCK cells, that overexpression of TM4SF10 reduced Fyn activity commensurate with reduced Fyn Y^421^ phosphorylation but with no change in Y^532^ phosphorylation, and that ADAP over expression did not appear to alter the suppressive effect of TM4SF10 on Fyn activity (Azhibekov et al. 2011). In addition, we also have previously shown in podocytes that PAN treatment reactivates TM4SF10 expression in podocytes (Azhibekov et al. 2011), such that PAN treatments cause endogenous TM4SF10 overexpression (example of TM4SF10 induction with PAN shown in Figure 6). In Figure 4, in the setting of TM4SF10 overexpression (i.e., PAN treatment) the Fyn activating pY^421^ levels decreased in both WT and KO podocytes. The Fyn inhibitory pY^532^ levels were unchanged in WT and KO podocytes, with or without PAN treatment. This indicates the absence of ADAP does not appear to influence Fyn activation, and is consistent with our prior studies in which ADAP overexpression also does not alter Fyn activation. Together, these studies indicate ADAP, either with or without of TM4SF10, does not alter the degree of Fyn activation in renal cells.

### ADAP effects on TM4SF10

We originally identified ADAP as a TM4SF10 binding partner, and we next examined the effect of ADAP on TM4SF10 in podocytes. Using PAN treatment to induce endogenous TM4SF10 expression, both WT and KO podocytes exhibited similar levels of TM4SF10 at cell‐cell contacts (Fig. 6). The TM4SF10 localized at cell‐cell junctions in both WT and KO podocytes, however, in ADAP KO podocytes there was a subtle alteration in the distribution of TM4SF10 at the cell contact. In ADAP KO podocytes, the typical “seam” of TM4SF10 at the point of cell contact was disrupted. Although TM4SF10 was present at cell‐cell contacts in KO cells, those contacts were not adjacent. This study indicated ADAP loss did not impact the induction of TM4SF10 expression during the injury‐repair response, but altered its positioning in the regenerating cell‐cell contact. Overall, these studies indicated ADAP likely does not have a critical role in cell adhesion, but may have a function in cytoskeleton events that may include the organization of cell‐cell contacts during injury‐repair processes.

## Discussion

The connection between ADAP and TM4SF10 in podocytes has been clarified in these studies. In our prior work, we established TM4SF10 is transiently expressed during early glomerular development but not in mature podocytes. Although normally absent in adult glomeruli, TM4SF10 is re‐expressed in podocytes during PAN‐induced injury/repair processes, resulting in suppressed Fyn kinase activity and the subsequent loss of cell protrusions (Bruggeman et al. 2007; Azhibekov et al. 2011). We have speculated the function of TM4SF10 re‐expression during injury/repair is to stabilize the damaged slit diaphragm that occurs with foot processes effacement (i.e., the loss of cell protrusions) by recreating a cadherin‐containing cell‐cell contact seen in podocyte precursors during development. In studies presented here, ADAP KO did not affect TM4SF10 re‐expression or suppression of Fyn activity associated with PAN treatment. However, ADAP KO phenocopied the TM4SF10 overexpression phenotype with the loss of the elaborate cell protrusions typical of cultured podocytes. We and others have previously shown ADAP is phosphorylated by Fyn and other Src family kinases (da Silva et al. 1997; Coppolino et al. 2001; Azhibekov et al. 2011), and phosphorylated ADAP binds Nck (Jones et al. 2006), a key adapter protein in podocytes that connects the slit diaphragm protein Nephrin to the actin nucleation complex, the Wiskott‐Aldrich syndrome family protein N‐WASP and actin‐related proteins ARP2/ARP3 (Grahammer et al. 2013). Thus, ADAP may be mediating the cytoskeletal events that control cells protrusions as a downstream consequence of TM4SF10 expression.

Although ADAP may have a role in actin cytoskeleton dynamics, we did not identify a clear role for ADAP in integrin function (i.e., cell adhesion) in podocytes. ADAP function is best described in T cells, where ADAP mediates integrin dependent events in the formation of the immunologic synapse (da Silva et al. 1997; Hunter et al. 2000; Peterson et al. 2001; Griffiths and Penninger 2002). We did not observe any alterations in integrin‐dependent cell attachment or formation of focal adhesions in the cultured ADAP KO podocytes. Although the attachment kinetics of ADAP KO podocytes was slower than WT podocytes, this may be a consequence of inefficient substrate tethering associated with the actin cytoskeleton remodeling needed for filopodia/lamellipodia formation. In podocytes, and similar to VAB‐9 in the worm epidermis (Simske et al. 2003), integrin‐dependent cell adhesion processes mediated by TM4SF10 and ADAP may have redundant or compensatory pathways.

Both the in vivo and in vitro effects of ADAP KO were subtle. We did not observe any obvious kidney development defects associated with ADAP absence. The long term in vivo studies found ADAP absence eventually had an impact on the maintenance of kidney structure and function. Similarly, the modest changes observed in vitro for the ADAP KO podocytes may be expected considering the renal phenotype in the KO mouse model required a year to develop sufficient pathological changes to impact function. However, ADAP function was either not critical for podocytes or else redundant mechanisms to counteract its absence were present. Considering the longevity of podocytes and their importance to kidney filtration, maintaining redundant systems for key cell functions would be logical.

In summary, our studies to understand TM4SF10‐ADAP function in the development, maintenance, and repair of podocytes has identified a key function to cytoskeleton rearrangements that may be related to filopodia and lamellipodia formation in foot process formation. The results of these studies are shedding new light on the molecular mechanisms controlling the formation of the podocyte foot process and slit diaphragm both during development but also in proteinuric kidney diseases characterized by foot process effacement and loss of the filtration barrier. The TM4SF10‐ADAP function in podocytes may have future therapeutic use in facilitating injury repair, or possibly blockade of injury, to protect or recover podocytes from irreparable loss or detachment. Because TM4SF10 is typically a silent gene in adulthood that is re‐expressed in acute injury processes, this molecule could be a target that could be exploited in novel therapy development.

## Conflict of Interest

The authors declare no conflict of interest.

## References

[phy213483-bib-0001] Azhibekov, T. A. , Z. Wu , A. Padiyar , L. A. Bruggeman , and J. S. Simske . 2011 TM4SF10 and ADAP interaction in podocytes: role in Fyn activity and Nephrin phosphorylation. Am. J. Physiol. Cell Physiol. 301:C1351–C1359. 21881001 10.1152/ajpcell.00166.2011 PMC3233801

[phy213483-bib-0002] Bruggeman, L. A. , S. Martinka , and J. S. Simske . 2007 Expression of TM4SF10, a Claudin/EMP/PMP22 family cell junction protein, during mouse kidney development and podocyte differentiation. Dev. Dyn. 236:596–605. 17195181 10.1002/dvdy.21052

[phy213483-bib-0003] Burbach, B. J. , R. Srivastava , R. B. Medeiros , W. E. O'Gorman , E. J. Peterson , and Y. Shimizu . 2008 Distinct regulation of integrin‐dependent T cell conjugate formation and NF‐kappa B activation by the adapter protein ADAP. J. Immunol. 181:4840–4851. 18802088 10.4049/jimmunol.181.7.4840 PMC2593878

[phy213483-bib-0004] Burbach, B. J. , R. Srivastava , M. A. Ingram , J. S. Mitchell , and Y. Shimizu . 2011 The pleckstrin homology domain in the SKAP55 adapter protein defines the ability of the adapter protein ADAP to regulate integrin function and NF‐kappaB activation. J. Immunol. 186:6227–6237. 21525391 10.4049/jimmunol.1002950 PMC3108501

[phy213483-bib-0005] Coppolino, M. G. , M. Krause , P. Hagendorff , D. A. Monner , W. Trimble , S. Grinstein , et al. 2001 Evidence for a molecular complex consisting of Fyb/SLAP, SLP‐76, Nck, VASP and WASP that links the actin cytoskeleton to Fcgamma receptor signalling during phagocytosis. J. Cell Sci. 114:4307–4318. 11739662 10.1242/jcs.114.23.4307

[phy213483-bib-0006] D'Agati, V. D. 2008 Podocyte injury in focal segmental glomerulosclerosis: lessons from animal models (a play in five acts). Kidney Int. 73:399–406. 17989648 10.1038/sj.ki.5002655

[phy213483-bib-0007] Dong, Y. , and J. S. Simske . 2016 Vertebrate Claudin/PMP22/EMP22/MP20 family protein TMEM47 regulates epithelial cell junction maturation and morphogenesis. Dev. Dyn. 245:653–666. 26990309 10.1002/dvdy.24404 PMC5322768

[phy213483-bib-0008] Garg, P. , R. Verma , and L. B. Holzman . 2007 Slit diaphragm junctional complex and regulation of the cytoskeleton. Nephron. Exp. Nephrol. 106:e67–e72. 17570942 10.1159/000101795

[phy213483-bib-0009] George, B. , and L. B. Holzman . 2012 Signaling from the podocyte intercellular junction to the actin cytoskeleton. Semin. Nephrol. 32:307–318. 22958485 10.1016/j.semnephrol.2012.06.002 PMC3438455

[phy213483-bib-0010] Grahammer, F. , C. Schell , and T. B. Huber . 2013 The podocyte slit diaphragm–from a thin grey line to a complex signalling hub. Nat. Rev. Nephrol. 9:587–598. 23999399 10.1038/nrneph.2013.169

[phy213483-bib-0011] Griffiths, E. K. , and J. M. Penninger . 2002 Communication between the TCR and integrins: role of the molecular adapter ADAP/Fyb/Slap. Curr. Opin. Immunol. 14:317–322. 11973129 10.1016/s0952-7915(02)00334-5

[phy213483-bib-0012] Huber, T. B. , and T. Benzing . 2005 The slit diaphragm: a signaling platform to regulate podocyte function. Curr. Opin. Nephrol. Hypertens. 14:211–216. 15821412 10.1097/01.mnh.0000165885.85803.a8

[phy213483-bib-0013] Hunter, A. J. , N. Ottoson , N. Boerth , G. A. Koretzky , and Y. Shimizu . 2000 Cutting edge: a novel function for the SLAP‐130/FYB adapter protein in beta 1 integrin signaling and T lymphocyte migration. J. Immunol. 164:1143–1147. 10640723 10.4049/jimmunol.164.3.1143

[phy213483-bib-0014] Jones, N. , I. M. Blasutig , V. Eremina , J. M. Ruston , F. Bladt , H. Li , et al. 2006 Nck adaptor proteins link nephrin to the actin cytoskeleton of kidney podocytes. Nature 440:818–823. 16525419 10.1038/nature04662

[phy213483-bib-0015] Kern, G. , and B. E. Flucher . 2005 Localization of transgenes and genotyping of H‐2 kb‐tsA58 transgenic mice. Biotechniques 38:38, 40, 42. 15679082 10.2144/05381BM03

[phy213483-bib-0016] Madhavan, S. M. , J. F. O'Toole , M. Konieczkowski , S. Ganesan , L. A. Bruggeman , and J. R. Sedor . 2011 APOL1 localization in normal kidney and nondiabetic kidney disease. J. Am. Soc. Nephrol. 22:2119–2128. 21997392 10.1681/ASN.2011010069 PMC3231786

[phy213483-bib-0017] Medeiros, R. B. , B. J. Burbach , K. L. Mueller , R. Srivastava , J. J. Moon , S. Highfill , et al. 2007 Regulation of NF‐kappaB activation in T cells via association of the adapter proteins ADAP and CARMA1. Science 316:754–758. 17478723 10.1126/science.1137895

[phy213483-bib-0018] Menasche, G. , S. Kliche , N. Bezman , and B. Schraven . 2007 Regulation of T‐cell antigen receptor‐mediated inside‐out signaling by cytosolic adapter proteins and Rap1 effector molecules. Immunol. Rev. 218:82–91. 17624945 10.1111/j.1600-065X.2007.00543.x

[phy213483-bib-0019] Mundel, P. , J. Reiser , B. A. Zuniga Mejia , H. Pavenstadt , G. R. Davidson , W. Kriz , et al. 1997 Rearrangements of the cytoskeleton and cell contacts induce process formation during differentiation of conditionally immortalized mouse podocyte cell lines. Exp. Cell Res. 236:248–258. 9344605 10.1006/excr.1997.3739

[phy213483-bib-0020] Patrakka, J. , and K. Tryggvason . 2010 Molecular make‐up of the glomerular filtration barrier. Biochem. Biophys. Res. Commun. 396:164–169. 20494132 10.1016/j.bbrc.2010.04.069

[phy213483-bib-0021] Pauker, M. H. , B. Reicher , S. Fried , O. Perl , and M. Barda‐Saad . 2011 Functional cooperativity between the proteins Nck and ADAP is fundamental for actin reorganization. Mol. Cell. Biol. 13:2653–2666. 10.1128/MCB.01358-10 PMC3133383 21536650

[phy213483-bib-0022] Peterson, E. J. , M. L. Woods , S. A. Dmowski , G. Derimanov , M. S. Jordan , J. N. Wu , et al. 2001 Coupling of the TCR to integrin activation by Slap‐130/Fyb. Science 293:2263–2265. 11567141 10.1126/science.1063486

[phy213483-bib-0023] Reeves, W. , J. P. Caulfield , and M. G. Farquhar . 1978 Differentiation of epithelial foot processes and filtration slits: sequential appearance of occluding junctions, epithelial polyanion, and slit membranes in developing glomeruli. Lab. Invest. 39:90–100. 682603

[phy213483-bib-0024] Rico, M. , A. Mukherjee , M. Konieczkowski , L. A. Bruggeman , R. T. Miller , S. Khan , et al. 2005 WT1‐interacting protein and ZO‐1 translocate into podocyte nuclei after puromycin aminonucleoside treatment. Am. J. Physiol. Renal Physiol. 289:F431–F441. 15798086 10.1152/ajprenal.00389.2004

[phy213483-bib-0025] Schmitt, R. , C. Jacobi , N. Susnik , V. Broecker , H. Haller , and A. Melk . 2009 Ageing mouse kidney–not always the SAME old story. Nephrol. Dial. Transplant. 24:3002–3005. 19474281 10.1093/ndt/gfp232

[phy213483-bib-0026] Shankland, S. J. , J. W. Pippin , J. Reiser , and P. Mundel . 2007 Podocytes in culture: past, present, and future. Kidney Int. 72:26–36. 17457377 10.1038/sj.ki.5002291

[phy213483-bib-0027] Shih, N. Y. , J. Li , V. Karpitskii , A. Nguyen , M. L. Dustin , O. Kanagawa , et al. 1999 Congenital nephrotic syndrome in mice lacking CD2‐associated protein. Science 286:312–315. 10514378 10.1126/science.286.5438.312

[phy213483-bib-0028] da Silva, A. J. , Z. Li , C. de Vera , E. Canto , P. Findell , and C. E. Rudd . 1997 Cloning of a novel T‐cell protein FYB that binds FYN and SH2‐domain‐containing leukocyte protein 76 and modulates interleukin 2 production. Proc. Natl Acad. Sci. USA 94:7493–7498. 9207119 10.1073/pnas.94.14.7493 PMC23849

[phy213483-bib-0029] Simske, J. S. , M. Koppen , P. Sims , J. Hodgkin , A. Yonkof , and J. Hardin . 2003 The cell junction protein VAB‐9 regulates adhesion and epidermal morphology in C. elegans. Nat. Cell Biol. 5:619–625. 12819787 10.1038/ncb1002

[phy213483-bib-0030] Srivastava, R. , B. J. Burbach , and Y. Shimizu . 2010 NF‐kappaB activation in T cells requires discrete control of IkappaB kinase alpha/beta (IKKalpha/beta) phosphorylation and IKKgamma ubiquitination by the ADAP adapter protein. J. Biol. Chem. 285:11100–11105. 20164171 10.1074/jbc.M109.068999 PMC2856986

[phy213483-bib-0031] Sylvester, M. , S. Kliche , S. Lange , S. Geithner , C. Klemm , A. Schlosser , et al. 2010 Adhesion and degranulation promoting adapter protein (ADAP) is a central hub for phosphotyrosine‐mediated interactions in T cells. PLoS ONE 5:e11708. 20661443 10.1371/journal.pone.0011708 PMC2908683

[phy213483-bib-0032] Wang, H. , and C. E. Rudd . 2008 SKAP‐55, SKAP‐55‐related and ADAP adaptors modulate integrin‐mediated immune‐cell adhesion. Trends Cell Biol. 18:486–493. 18760924 10.1016/j.tcb.2008.07.005 PMC3512129

[phy213483-bib-0033] Yabuki, A. , S. Tanaka , M. Matsumoto , and S. Suzuki . 2006 Morphometric study of gender differences with regard to age‐related changes in the C57BL/6 mouse kidney. Exp. Anim. 55:399–404. 16880688 10.1538/expanim.55.399

[phy213483-bib-0034] Yang, H. C. , Y. Zuo , and A. B. Fogo . 2010 Models of chronic kidney disease. Drug Discov Today Dis Models 7:13–19. 21286234 10.1016/j.ddmod.2010.08.002 PMC3030258

[phy213483-bib-0035] Yu, A. S. 2015 Claudins and the kidney. J. Am. Soc. Nephrol. 26:11–19. 24948743 10.1681/ASN.2014030284 PMC4279745

